# How deep is deep enough for RNA-Seq profiling of bacterial transcriptomes?

**DOI:** 10.1186/1471-2164-13-734

**Published:** 2012-12-27

**Authors:** Brian J Haas, Melissa Chin, Chad Nusbaum, Bruce W Birren, Jonathan Livny

**Affiliations:** 1 Genome Sequencing and Analysis Program, The Broad Institute of MIT and Harvard, Cambridge, MA, 02142, USA; 2 Channing Laboratory, Brigham and Women’s Hospital, Harvard Medical School, Boston, MA, 02115, USA

## Abstract

**Background:**

High-throughput sequencing of cDNA libraries (RNA-Seq) has proven to be a highly effective approach for studying bacterial transcriptomes. A central challenge in designing RNA-Seq-based experiments is estimating *a priori* the number of reads per sample needed to detect and quantify thousands of individual transcripts with a large dynamic range of abundance.

**Results:**

We have conducted a systematic examination of how changes in the number of RNA-Seq reads per sample influences both profiling of a single bacterial transcriptome and the comparison of gene expression among samples. Our findings suggest that the number of reads typically produced in a single lane of the Illumina HiSeq sequencer far exceeds the number needed to saturate the annotated transcriptomes of diverse bacteria growing in monoculture. Moreover, as sequencing depth increases, so too does the detection of cDNAs that likely correspond to spurious transcripts or genomic DNA contamination. Finally, even when dozens of barcoded individual cDNA libraries are sequenced in a single lane, the vast majority of transcripts in each sample can be detected and numerous genes differentially expressed between samples can be identified.

**Conclusions:**

Our analysis provides a guide for the many researchers seeking to determine the appropriate sequencing depth for RNA-Seq-based studies of diverse bacterial species.

## Background

In recent years, high throughput sequencing of cDNA libraries (RNA-Seq) has emerged as a powerful technology for profiling gene expression, discovering previously unannotated genes, and mapping transcriptome architecture in a wide variety of bacterial species [1-11]. RNA-Seq offers several advantages over hybridization-based approaches such as microarrays, including a markedly higher sensitivity for low abundance transcripts, single nucleotide resolution of transcript boundaries, and the means to profile gene expression in strains for which genome sequences and/or gene annotations are not available [12,13]. The steadily decreasing cost of sequencing, the growing number of and accessibility to high-throughput sequencing facilities, and the recent development of publicly available bioinformatic tools for RNA-Seq data analysis have made RNA-Seq an increasingly attractive and popular method for studying bacterial transcriptomes.

The relative abundances of individual transcripts in a bacterial transcriptome can differ by several orders of magnitude. In order to generate comprehensive transcriptome profiles using RNA-Seq one must therefore obtain a sufficiently large number of reads to detect those biologically relevant transcripts that comprise a relatively small proportion of the cDNA library. Detection and quantification of low abundance transcripts by RNA-Seq can be enhanced in two main ways. First, the total number of reads per library can be increased. Second, the proportion of reads representing rare transcripts can be increased by depleting abundant transcripts from total RNA and/or depleting cDNAs representing these abundant transcripts from cDNA libraries. This is often achieved by targeted removal of ribosomal RNA (rRNA), which comprises 80-95% of bacterial transcriptomes, from total RNA prior to cDNA library construction [14,15].

For many RNA-Seq-based projects, the budget for sequencing costs, and thus the total number of reads that can be obtained, is constrained. Thus, researchers designing RNA-Seq experiments must often determine the correct balance between sequencing depth (the number of reads per sample) and breadth (the number of samples sequenced). For some applications of RNA-Seq such as transcriptome mapping and annotation, the ability to detect rare transcripts is critical, and approaches such as the ones described above for increasing the total number of biologically relevant reads obtained per sample play a central role. For other applications of RNA-Seq breadth can often be more important than depth. Specifically, for experiments focused on comparing gene expression among various strains and/or growth conditions, the inclusion of more strains, timepoints, biological replicates, and/or growth conditions may be worth the tradeoff of lower depth per sample, as it may provide additional biological insights and/or statistical confidence that is more valuable than the ability to detect low abundance transcripts in each sample.

In recent years, methods for incorporating barcoded adapters into cDNA libraries have been developed that allow reads derived from up to several dozen samples to be sequenced in the same lane [16]. This approach, known as multiplexing, enables researchers to flexibly vary the number of samples sequenced per lane and thus obtain the desired balance between the number of samples included and the number of reads obtained per sample, in particular when number of lanes of sequencing is budget limited. However, the extent to which biologically relevant information is gained or lost as sequencing depth is varied has not been systematically examined. To address this we have generated and analyzed a variety of RNA-Seq datasets to determine the number of reads needed to saturate the transcriptome of *E. coli* and examined how reducing sequencing depth affects the ability to detect and quantify transcripts both within and between samples in diverse bacterial species.

## Results

### Ultra-deep sequencing of the *E. coli* transcriptome

Previous studies have suggested that accurate quantification of > 95% of transcripts in a mammalian cell line (including splice junction level quantification) requires ~700 million reads [17]; however, no estimate of the number of reads needed to approach saturation of a bacterial transcriptome has been reported. To address this question, we isolated total RNA from a log phase culture of *Escherichia coli* K-12 which was then depleted of rRNA using the RiboZero kit (Epicentere), converted to a strand-specific Illumina cDNA library as described [14], and sequenced in one lane of Illumina HiSeq. This produced a dataset of more than 306 million total reads aligning to the *E. coli* K-12 genome. Over 97% of these reads corresponded to properly mapped paired end reads, i.e. those corresponding to reads derived from opposite ends of the same cDNA mapping no more than 450 base pairs apart on the genome (the approximate maximum size of cDNAs in the library – see Methods). Properly mapped paired end reads were resolved into a single fragment by filling in the gap between them (if any). For pairs of reads that was not properly mapped, one read was discarded and the remaining reads along with unpaired reads were each treated as independent fragments. In total this dataset contained approximately 156 million aligned fragments with an average length of 159 nucleotides. rRNA depletion in this sample was nearly complete, with less than 0.15% of fragments aligning to rRNA-encoding genes (Additional file 1: Table S1).

**Figure 1:**
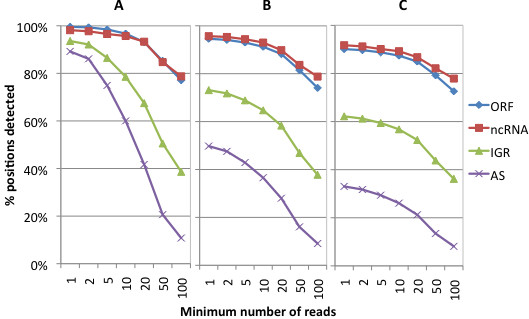
**Coverage of the *E. coli *K-12 genome by ultra-deep RNA-Seq data.** Annotation of genomic positions as antisense ORF, non-coding RNA (ncRNAs) intergenic (IGR), and antisense to ORFs or ncRNAs (AS) was based on gene annotations in the RefSeq and Rfam databases. Positions on the opposite strand of genes were annotated as antisense only if no other genes were annotated at those positions. **A**) without gDNA subtraction. **B**) with 0.5% gDNA subtraction. **C**) with 1% gDNA subtraction.

The proportion of annotated ORFs represented in this dataset was very high, with all but 2 of 4149 ORFs annotated in RefSeq covered by at least 1 fragment (Additional file 2: Table S2). Coverage of the genome also approached saturation, with at least 1 fragment mapping to over 94% of strand-specific genomic positions. Importantly, the density of this coverage varied markedly among different regions of the genome (Figure 1A). For example, while 96% of bases within annotated ORFs were detected by 10 or more fragments, only 60% of bases in regions antisense to annotated ORFs were detected above this cutoff. Similarly, the density of coverage was relatively high for genes encoding non-coding RNAs (ncRNAs) and relatively low in intergenic regions (Figure 1A).

As shown in Figure 1A, a surprisingly high proportion of antisense and intergenic positions were covered by at least 1 fragment. We reasoned that this could be due to limitations in the method used to maintain strand specificity in our libraries [18,19]. In this method, dUTPs are incorporated only into the second strand of cDNAs during cDNA synthesis and these dUTPs are then excised prior to library amplification, ensuring that only the first cDNA strand is efficiently amplified. Incomplete incorporation and/or excision of dUTPs would presumably lead to low levels of antisense fragments corresponding to the second strand of cDNAs. To assess the level of second strand contamination in our samples, we compared the average fragment coverage on the sense and antisense strands of each annotated ORF with the expectation that this coverage should be somewhat correlated if second strand removal was incomplete. As shown in Additional file 3: Figure S1, there was very little positive correlation (R^2^ = 0.0004) between the fragment coverage of sense and antisense strands, even among highly expressed genes. In contrast, the correlation in the coverage of ORF sense and antisense strands was much higher (R^2^=0.83) when a similar rRNA-depleted *E. coli* cDNA library was not subjected to dUTP excision prior to amplification and sequencing. Thus, incomplete strand specificity in our libraries does not seem to have contributed significantly to the observed high coverage of antisense positions.

Another explanation for the high coverage of antisense and intergenic positions observed is that a much higher proportion *E. coli* genome is transcribed than is suggested by current gene annotations. Indeed, several recent studies have demonstrated widespread transcription from the antisense strand of protein-encoding genes in diverse bacteria [20-23]. While in some cases these antisense transcripts have been shown to play important regulatory functions, two recent studies in *Bacillus subtilis* and *E. coli* K-12 suggest that many antisense RNAs derive from spurious transcription initiation or incomplete transcription termination and may not be functionally relevant [24,25]. Thus many of the fragments aligning to intergenic regions of the genome may correspond to non-specific transcription initiation or leaky transcription termination of upstream genes. Other sequences from intergenic regions may be derived from previously unannotated ncRNAs. Recent studies suggest the prevalence of ncRNA genes has likely been underestimated, even in well-studied bacteria such as *E. coli* K-12 [7,26].

Finally, the nearly complete RNA-Seq read coverage of the genome could also reflect contamination of our cDNA libraries with a low amount of *E. coli* genomic DNA (gDNA). While total RNA was subjected to 2 rounds of DNase treatment and no gDNA was detected following 40 rounds of PCR prior to cDNA synthesis, it is possible that removal of gDNA from our total RNA was not complete. Similarly, reagents used after DNase treatment in library construction may also have introduced low amounts of *E. coli* gDNA contamination.

Taken together our findings suggest that a sequencing depth of 156 million fragments is sufficient to saturate the *E. coli* K-12 transcriptome but also yields numerous fragments aligning to very rare and potentially non-functional transcripts and/or to low-level contaminants introduced during library construction.

### Genome coverage of RNA-Seq data after background subtraction

While read coverage of annotated *E. coli* genes was nearly complete in the 156M read dataset, the possibility of gDNA contamination raised concern that some of these genes were not actually transcribed. To better estimate the proportion of *E. coli* genes transcribed under the conditions tested, we devised an algorithm to subtract potential gDNA background from our RNA-Seq dataset based on the assumption that, unlike reads corresponding to cDNAs, the alignment of reads corresponding to gDNA would be uniformly distributed across the *E. coli* genome. As shown in Figure 1B and 1C, background subtraction assuming 0.5% or 1% gDNA contamination led to relatively modest decreases in ORF and ncRNA coverage but to significant drops in coverage of IGR and AS positions. Indeed, after applying a 1% background subtraction, only 33% and 62% of AS and IGR positions were covered at saturation, respectively, compared to 90% and 92% of ORF and ncRNA position, respectively. While the actual extent of gDNA contamination is difficult to ascertain, the results of our PCR screen prior to cDNA synthesis suggest it is unlikely to be as high as 1%. Yet even with this high level of subtraction, at least 1 and 10 reads aligned to 98% and 95% of annotated ORFs, respectively, suggesting that a very high proportion of annotated *E. coli* genes are expressed at least at low levels during exponential growth in rich media.

Of the 100 ORFs to which no reads aligned following 1% subtraction, several are near the minimum size cutoff of cDNAs efficiently maintained during library construction. These include 4 of the 5 *ibs* toxic membrane proteins that may indeed not be expressed under normal growth conditions [27] (Additional file 2: Table S2). Importantly, ORFs annotated as “predicted proteins” or encoded within annotated prophages were enriched more than 2- and 4-fold, respectively, among the undetected ORFs. Moreover, many undetected ORFs were clustered in known operons, including 5 of 7 ORFs in the *rut* operon involved in pyrimidine degradation [28], 5 of 6 ORFs in the *cit* operon encoding components of an inactive citrate lyase [29], and 8 of 15 ORFs in the *phn* operon required for use of phosphonate and phosphite as phosphorous sources [30] (Additional file 2: Table S2). Some of the 100 ORFs not represented in our RNA-Seq data have been shown to be expressed in other studies conducted under different growth conditions, suggesting the transcription of these genes is highly repressed and/or the half-lives of these transcripts is very short during exponential growth of *E. coli* K-12 in LB medium.

### Effect of ribosomal RNA depletion on RNA-Seq transcriptome profiles

We next assessed to what extent rRNA depletion increases detection of low expressed transcripts by RNA-Seq. To this end, we constructed another Illumina library derived from the same total RNA used to produce the initial rRNA-depleted dataset and sequenced this library in a single Illumina HiSeq lane. While the depleted and undepleted libraries yielded a similar number of total fragments, 82% number of fragments in the undepleted sample aligned to rRNAs and the number of fragments aligning to ORFs in this sample was more than 8-fold lower than in the depleted sample.

**Figure 2:**
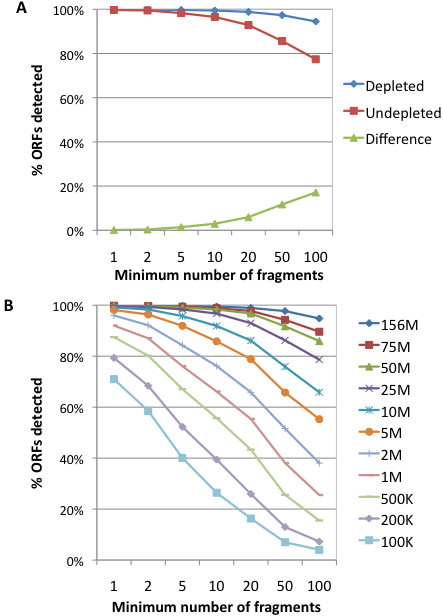
**Coverage of annotated *E. coli *K-12 ORFs by ultra-deep RNA-Seq data.** In each plot, the X-axis denotes the minimum threshold of fragments/ORF **A**) Coverage of ORFs by data derived from rRNA-depleted and undepleted samples. **B**) Coverage of ORFs by full and sampled datasets of the rRNA-depleted sample.

As expected, the proportion of annotated ORFs detected was higher in the depleted than the undepleted samples (Figure 2A). However, even in the undepleted sample, at least one fragment mapped to over 99% of annotated ORFs, and over 96% of annotated ORFs were associated with 20 or more fragments. Moreover, the subset of ORFs detected with a minimum of 10 fragments per ORF was only 2% lower in the undepleted sample. Thus, in a dataset containing enough fragments to saturate the *E. coli* transcriptome, the lack of rRNA depletion greatly reduced the number of mRNA-derived fragments obtained but led to only a relatively modest decrease in the proportion of annotated *E. coli* ORFs detected.

### Effect of decreased sequencing depth on RNA-Seq transcriptome coverage in *E. coli*

To systematically assess how decreasing fragment counts per sample affects the comprehensiveness of gene expression profiles, we developed scripts that randomly sampled our 156 million fragment rRNA-depleted *E. coli* RNA-Seq dataset to create datasets with decreasing numbers of fragments. The ORF and genome coverage provided by these datasets was then quantified and compared. To ensure our sampling approach accurately simulated multiplexing, we re-sequenced the *E. coli* cDNA library, this time multiplexed with 11 unrelated libraries in the same HiSeq lane, producing a dataset with approximately 15 million total fragments. Importantly, both the levels of genome coverage and the number of fragments per ORF in this dataset correlated very well (R^2^ > 0.99) with those of a dataset of 15 million fragments sampled from the 156 million fragment dataset.

As shown in Figure 2B, reducing the number of fragments led to a decrease in the proportion of annotated ORFs to which 1 or more fragments aligned. However, this decrease was often relatively small compared to the reduction in the number of fragments. For example, decreasing the number of fragments over 15-fold from 156 to 10 million fragments led to only a 3% and 7% loss in the number of ORFs detected with more than 5 and 10 fragments, respectively. Indeed, even with only 2 million fragments, 96% and 84% of ORFs were covered by at least 1 fragment and 5 fragments, respectively.

**Figure 3:**
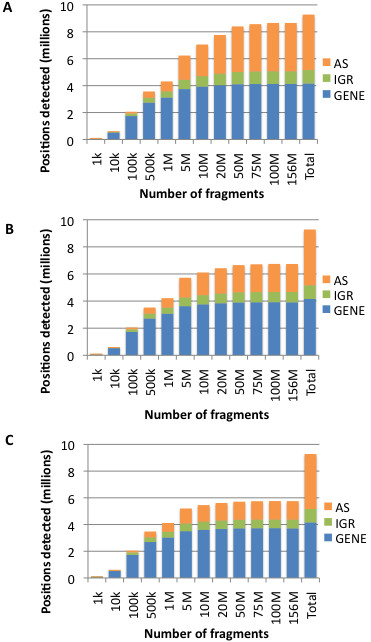
**Coverage of *E. coli *K-12 genome as sequencing depth increases.** Annotations of genomic positions was conducted as described in the Figure 1 legend. The bar labeled “Total” represents all positions in the *E. coli* K-12 genome. **A**) without gDNA subtraction. **B**) with 0.5% gDNA subtraction. **C**) with 1% gDNA subtraction.

As shown in Figure 3A, positions within annotated genes were nearly saturated by 50 million fragments, and only relatively incremental increases in annotated gene coverage were obtained above 10 million fragments. A similar trend was observed in intergenic positions. As the number of fragment continues to increase beyond 50 million, nearly all new positions detected were within antisense regions of the genome, many of which, as discussed above, may correspond to non-functional spurious transcripts or gDNA contamination. Indeed, in the background subtracted datasets, very few new positions were detected in any category in datasets with more than 50 million fragments (Figure 3B and 3C). Taken together, these findings suggest that 50 million non-rRNA fragments yield nearly complete coverage of biologically relevant *E. coli* transcripts expressed during log phase growth in LB. Moreover, they suggest that vast majority of the *E. coli* transcriptome can be detected under this growth condition even with datasets of only 5-10 million non-rRNA fragments.

### Effect of decreased sequencing depth on RNA-Seq transcriptome coverage in *M. tuberculosis* and *V. cholerae*

**Figure 4:**
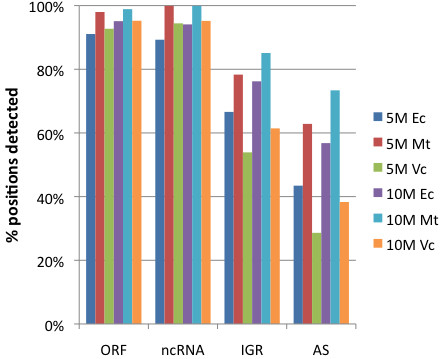
**Coverage of various bacterial genomes by RNA-Seq data with varying sequencing depth.** Annotations of genomic positions was conducted as described in the Figure 1 legend. 5M and 10M denote RNA-Seq databases with 5 and 10 million non-rRNA fragments, respectively. Ec, Mt, and Vc correspond to *E. coli* K-12, *Mycobacterium tuberculosis*, and *Vibrio cholerae*, respectively.

The regulatory networks governing gene expression can diverge significantly among different bacteria. Moreover, patterns of gene expression can vary dramatically among different growth conditions. To assess whether the relationship between sequencing depth and transcriptome coverage described above extends beyond log-phase *E. coli* K-12 cultures growing in LB, we repeated the analysis above with RNA-Seq data derived from log phase LB cultures of *Mycobacterium tuberculosis* (Figure 4), a species whose GC content, gene content and organization, and physiology are significantly diverged from those of *E. coli*. Importantly, similar levels of coverage of annotated ORFs and ncRNAs were seen in these *M. tuberculosis* datasets containing 5 and 10 million non-rRNA fragments (Figure 4). We also analyzed RNA-Seq datasets containing 5 and 10 million non-ribosomal fragments derived from log phase cultures of *Vibrio cholerae* growing in M9 minimal medium [3] and found similar levels of gene coverage, though coverage of antisense and intergenic regions in these data was somewhat lower (Figure 4). These results suggest that a sequencing depth of 5-10 million non-rRNA fragments enables profiling of the vast majority of transcriptional activity in diverse species grown under diverse culture conditions.

### Using RNA-Seq to identify differentially expressed genes: how important is depth?

In addition to its utility in profiling the transcriptome of a single strain of interest, RNA-Seq is also a powerful tool for comparing gene expression among different strains and/or growth conditions. A recent study by Tarazona *et al.* examined the relationship between sequencing depth and the reliable identification of changes in gene expression in human RNA-Seq data [31] but to date no similar analysis has been conducted for bacterial RNA-Seq data. To assess how changes in sequencing depth influence RNA-Seq-based analysis of differential gene expression in bacteria, we sequenced rRNA-depleted total RNA isolated from LB cultures of *E. coli* O157:H7 strain EDL933 (from hereon referred to as EDL933) at the late exponential and early stationary phases. cDNA libraries corresponding to 2 biological replicates for each time point were subjected to multiplexed sequencing using Illumina HiSeq to yield 25-30 million fragments per sample. Data between biological replicates for each time point was were extremely well correlated (R^2^ of fragments/ORF = 0.99). To examine the impact of having fewer fragments on the results of differential expression analysis, we scaled down the counts of fragments per gene from each dataset while retaining the original values of relative gene expression.

**Figure 5:**
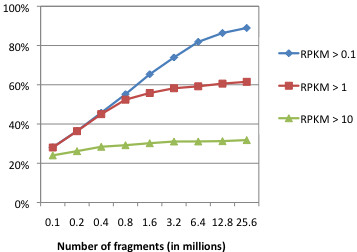
**Effect of sequencing depth on the detection of transcripts in two independent biological replicates.** The percent of all annotated ORFs detected by RNA-Seq above the indicated RPKM thresholds in both replicates of EDL933 exponential phase cultures.

We first used these sampled datasets to determine how changes in sequencing depth influenced the detection of transcripts in two independent biological replicates. As shown in Figure 5, the total number of transcripts detected in both replicates rose significantly as depth was increased, particularly among lowly expressed genes. Importantly, these increases began reaching an asymptote around 13 million fragments, suggesting that additional depth beyond this point did relatively little to increase either the number or percent of all genes detected in both biological replicates.

**Figure 6:**
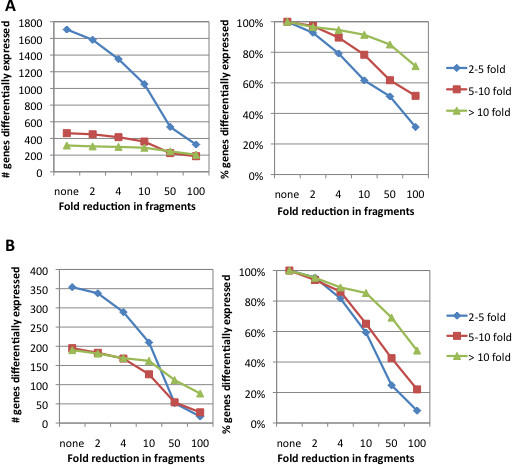
**Effect of decreased sequencing depth on detection of differentially expressed genes by RNA-Seq.** Differentially expressed genes were identified by DESeq with P < 10^-3^. **A**) Comparison of EDL933 gene expression in exponential and stationary phase. The total number of aligned non-rRNA fragments in these datasets ranged from 25-30 million. **B**) Comparison of *V. cholerae* gene expression in minimal media and the rabbit cecum. The total number of aligned non-rRNA fragments in these datasets ranged from 4-6 million.

We next analyzed the full and sampled datasets with DESeq, a variance-analysis package that uses a model based on the negative binomial distribution to infer statistically significant differences in gene-expression from RNA-Seq data [32]. Based on the counts of gene-mapped fragments derived from the full RNA-Seq data set of ~25 million fragments per sample, DESeq identified 2486 genes (corresponding to 45% of all annotated EDL933 genes) as being at least 2-fold up- or down-regulated (P < 1×10^-3^). As shown in Figure 6A, reductions in sequencing depth correlated with a decrease in the number of genes identified as differentially expressed below this P-value cutoff. As expected, the effect of decreased depth was most marked for genes whose differential abundance between the two growth phases was relatively small (Figure 6A). For example, a 10-fold decrease in depth resulted in a loss of 38% of genes 2-5-fold differentially expressed but only 9% of genes whose differential expression was greater than 10-fold. However, even when the depth was reduced to 2.5-3 million fragments in each dataset, 1704 genes were identified as differentially regulated more than 2-fold with P < 1×10^-3^. Our findings indicate that when data from well-correlated biological replicates are included, 2-3 million fragments per sample enable a significant number of genes differentially expressed by 2-fold or more to be identified with high statistical significance.

The ability to reliably identify differentially expressed genes by RNA-Seq is affected by a variety of factors aside from total sequencing depth that can vary significantly from one experiment to another, including the number of biological replicates included and the variation between them, the average abundance of differentially expressed genes, and the magnitude of their differential expression under the conditions tested. We therefore repeated the analysis above with RNA-Seq data that were distinct in several ways from the EDL933 data. Specifically, these data were derived from *V. cholerae* growing in M9 minimal medium or isolated from the cecal fluid of 2 orally infected infant rabbits [3]. Moreover, the correlation between the 2 rabbit samples was much lower than for the EDL933 in vitro samples (R^2^=0.69). Finally, the total number of non-rRNA fragments for these datasets was between 4 and 6 million, significantly less than in the EDL933 datasets.

Despite these numerous differences, the impact of reducing the number of fragments in the *V. cholerae* and EDL933 datasets on the ability to detect differentially expressed genes was very similar (Figure 6B). Importantly, as we observed in the analysis of the EDL933 data, numerous genes were identified as differentially expressed by at least 2-fold (P < 1×10^-3^) even with a reduction of fragments to 2-3 million per sample. These included all 16 of the major *V. cholerae* colonization or virulence factors identified as induced in the rabbit when the full datasets were compared [3]. Strikingly, the differential expression of all but one of these major colonization and virulence factors was detected (P < 1×10^-3^) even when the total number of non-rRNA fragments was reduced 100-fold to 40,000-60,000 total fragments. While it is not possible to accurately simulate how changes in depth will affect RNA-Seq comparative gene expression analyses in all cases, our findings indicate that in diverse species and growth conditions and even with relatively low correlation between biological replicates, 2-3 million fragments per sample enable a significant number of genes differentially expressed by 2-fold or more to be identified with high statistical significance.

## Discussion

We have conducted a systematic analysis of how changes in sequencing depth affect analysis of bacterial RNA-Seq data, both for profiling gene expression in a single sample and for comparing gene expression among different strains and/or growth conditions. Our findings suggest that 5-10 million non-rRNA fragments are sufficient to detect all but a few of the most low expressed genes in diverse bacteria growing under a variety of conditions. Moreover, we found that when the number of non-rRNA fragments in *E. coli* exceeds 50 million, detection of biologically relevant transcripts all but ceases and much of the additional coverage gained appears to represent very rare transcriptional events and/or gDNA contamination. We also found that when RNA-Seq data from biological replicates is available, differential expression of numerous genes can be detected with high statistical significance even when the number of fragments per sample is reduced to 2-3 million.

The optimal sequencing depth for an RNA-Seq based study will vary considerably based on the scientific objective of that study. For applications requiring a comprehensive transcriptome profile, coverage exceeding 10 million fragments per sample may be needed, with the understanding that increasing depth can lead to detection of sequences that may not represent *bona fide* transcripts. Alternatively, the number and diversity of growth conditions included in the analysis can be increased with the expectation that, while the number of reads per sample will be decreased, numerous transcripts whose abundance is low under one condition will be more highly expressed and thus easier to detect under another condition. For applications aimed at discovery of a relatively small number of previously unannotated genes to be subjected to experimental validation and/or functional characterization, lower sequencing depth can provide sufficient sensitivity. Indeed, a depth of 4 million non-rRNA fragments was sufficient for identification of several dozen previously unannotated ncRNAs in *V. cholerae*[3]. Similarly, even with only 25,000-30,000 non-rRNA fragments per sample we were able to identify 184 annotated genes in EDL933 whose abundance differed more than 2-fold between late exponential and early stationary phases (P < 1×10^-5^). Thus, our findings suggest that for many RNA-Seq based studies in bacteria, the number of fragments needed to profile gene expression in a single rRNA-depleted sample isolated from a bacterial monoculture is far less than that produced in a single Illumina HiSeq lane. Indeed, our findings suggest that at a certain point increased sequencing depth may actually be detrimental to the accurate mapping of biologically relevant transcripts, yielding reads that likely represent contaminants in the cDNA library or the products of spurious transcriptional events.

A HiSeq lane typically produces about 150 million paired end reads under current run conditions. Thus, multiplexing 15-30 samples per lane will yield the 5-10 million reads per sample that are sufficient for most applications of bacterial RNA-Seq. Indeed, our findings suggest that for studies of differential gene expression, even significantly higher levels of multiplexing result in relatively modest decreases in sensitivity. For these types of studies, the added biological information provided by the inclusion of more strains, growth conditions, and/or biological replicates may outweigh this loss of sensitivity for detecting transcriptional changes in each pairwise comparison of samples. Our findings also suggest that for studies in which only a few samples are to be sequenced in a single lane, a sufficient number of reads may be obtained for samples that are not depleted of rRNA and thus the time and cost associated with rRNA-depletion may not be justified. Finally, for studies involving only one or two samples, such as pilot or proof-of-principle experiments, lower throughput platforms such as Illumina MiSeq platform may be more appropriate than the HiSeq platform. MiSeq yields only about 7.5 million paired end reads per lane with a only a slightly lower reagent cost than a lane of HiSeq but produces data in a fraction of the time needed for a HiSeq run, making it a good option for those seeking to quickly obtain profiles of gene expression in only a few rRNA-depleted samples.

The analysis we conducted was largely limited to data derived from single bacterial strains grown in culture. However, RNA-Seq is increasingly being used to study the transcriptomes of bacteria growing in animal hosts and/or as part of complex bacterial communities. Samples isolated from animal models are often contaminated with a large amount of host RNA. In RNA derived from microbial communities, transcripts corresponding to particular strains of interest will often be greatly outnumbered by those expressed by the numerous other members of the community. Thus, in RNA-Seq data representing mixed samples, the number of reads corresponding to transcripts of interest can be orders of magnitude lower than in data derived from a homogeneous bacterial culture. Using RNA-Seq to unravel the dynamics of bacterial gene expression in these complex and biologically relevant samples will therefore require significantly greater sequencing depth per sample, a robust depletion of bacterial rRNA, host rRNA, and host mRNA, and/or enrichment for transcripts of interest through methods such as hybrid capture.

## Conclusion

We have conducted a systematic analysis of how changes in sequencing depth influence the profiling and comparison of transcriptomes by RNA-Seq in diverse bacterial species and growth conditions. Our findings provide a guide for determining the appropriate sequencing depth for a wide variety of RNA-Seq-based studies of bacterial gene expression.

## Methods

### RNA extraction and processing

RNA was isolated by incubation by TRIzol (Invitrogen) followed by passage through Direct-zol columns (Zymo Research). Isolation of *M. tuberculosis* RNA included bead beating during incubation with TRIzol [33]. Total RNA was depleted of ribosomal RNA using the Ribo-Zero rRNA Removal Gram-negative Kit (for *E. coli* and EDL933) and Gram-negative Kit (for *M. tuberculosis*) (Epicentre) according to the manufacturer’s protocol. mRNA-enriched RNA isolated using Zymo RNA Clean & Concentrator columns (Zymo Research) and treated with DNase using the TURBO DNA-free kit (Ambion) according to the manufacturer's protocol. The RNA was then fragmented in a reaction with 5X Fragmentation Buffer (Affymetrix) heated at 80°C for 6 minutes and purified using the Zymo RNA Clean & Concentrator columns (Zymo Research).

### cDNA synthesis

Unless otherwise indicated, all reagents in this section were obtained from Invitrogen. For first strand cDNA synthesis, RNA was incubated with random hexamers at 70°C for 10 minutes and then chilled on ice. The primer and RNA template mix was then added to 5X FS Buffer, 0.1 M DTT, 10 mM dNTP mix, Actinomycin D (Sigma-Aldrich), Superase-in (Ambion), and SuperScript III. This reaction was incubated at 25°C for 10 minutes and at 55°C for 1 hour, then chilled for 5 minutes on ice and cleaned up using Zymo RNA Clean & Concentrator (Zymo Research). The second strand cDNA synthesis reaction contained the product of the first strand synthesis reaction, 5X FS Buffer, 5X SS Buffer, 0.1 M DTT, 10 mM dUTP mix (Affymetrix/USB), RNase H, DNA Ligase (NEB), and *E. coli* DNA polymerase I (NEB). This reaction was incubated at 16°C for 2 hrs then placed on ice and terminated with 10 ul of 0.5 M EDTA. cDNA was then isolated from this reaction using the MinElute PCR Purification Kit (Qiagen).

### Illumina library construction and sequencing

cDNA fragments were end-repaired and phosphorylated, followed by adenylation of 3′ends and adapter ligation as described [34] with the exception of replacing standard paired end adapters with forked adapters containing unique 8 base index sequences. Samples were gel size-selected for 150-450bp fragment size (4% agarose, 85V, 3 hours.) Size-selected adaptor ligated cDNA was preincubated with 1 ml Uracil-Nglycosylase (Applied Biosystems) at 37°C for 15 minutes to remove uracils from the second cDNA strand. Following incubation at 95°C for 5 minutes, each sample underwent 18 cycles of PCR in 4 duplicate reactions. Each set of 4 reactions was then combined and purified using MinElute columns (Qiagen). Purified libraries were profiled using the Agilent Bioanalyzer and sequenced using the Illumina Hi-Seq platform to yield 76-101b paired end reads.

### RNA-Seq data analysis

Reads were aligned to RefSeq reference genomes (see Additional file 1: Table S1) using BWA [35] version 5.9. Gene annotations were obtained from RefSeq and Rfam [36]. The overall fragment coverage of genomic regions corresponding to features such as ORFs and rRNAs was conducted as described [3].

In calculating the number of fragments aligning to each feature, the paired-end strand-specific RNA-Seq reads were assigned to these features based on their overlapping genomic coordinates and strand orientation using a custom PERL script. Counts of RNA-Seq fragments were computed for each feature based on the paired-read mappings. Fragments aligning to the DNA strand opposite from the transcribed orientation of corresponding annotated features were classified and counted as antisense. In the minority of cases where only one read of a pair aligned to the genome, the entire fragment was assigned to the overlapping feature. Where each paired read of individual fragments aligned to different features, each feature was assigned a partial fragment count corresponding to 1/(number of mapped features). Differentially expressed genes were identified using the feature-assigned fragment counts for each replicate as input to the DESeq software [32].

Genome sequence coverage by RNA-Seq alignments was computed using a custom PERL script, where the strand-specific nucleotide coverage (C) was incremented at each nucleotide position spanned by a read or across the range covered by the boundaries of an RNA-Seq fragment inferred from a pair of properly mated paired end reads. Background subtraction assuming a given percent of genomic DNA contamination (pctBkg) was performed as follows. The total strand-specific coverage was computed by summing strand-specific nucleotide-level coverage (Csum) observed across the genome. The expected nucleotide-level coverage due to genomic DNA contamination (Cbkg) was computed as:

$$\text{Cbkg}=\text{Csum}*\left(\text{pctBkg}/1000\right)$$

The effective nucleotide-level background-subtracted coverage (Ceff) values were computed as follows:

$$\begin{array}{l} \text{Ceff}=\{\;\left(C-\text{Cbkg}<=0\right):0,\\ \;\left(C-\text{Cbkg}>=1\right)\;:\;\text{floor}\left(C-\text{Cbkg}\right),\\ \;\left(0\;<C-\text{Cbkg}<1\right)\;:1\\ \;\text{with}\;\text{probability}\;\left(C-\text{Cbkg}\right)\;\text{else}\;0\} \end{array}$$

## Abbreviations

cDNA: Complementary DNA synthesized from RNA; RNA-Seq: High throughput sequencing of cDNA libraries; ORF: Open reading frame; ncRNA: Non-coding RNA; rRNA: Ribosomal RNA; tRNA: Transfer RNA; gDNA: Genomic DNA.

## Competing interests

The author’s declare that they have no competing interests.

## Authors’ contributions

MC constructed *E. coli* and *M. tuberculosis* cDNA Illumina libraries. BJH and JL analyzed data. JL, BWB, and CN directed the project and coordinated the research. JL wrote the paper with input from BJH, BWB, and CN. All authors read and approved the final manuscript.

## Supplementary Material

Additional file 1 **Table S1.** Refseq accession numbers for strains included in this study. Click here for file

Additional file 2 **Table S2.** Reads per annotated ORF in 156M fragment data set before and after background subtraction. Click here for file

Additional file 3 **Figure S1.** Correlation of coverage of the sense and antisense strands of annotated ORFs. Click here for file

## References

[B1] Cho BK Zengler K Qiu Y Park YS Knight EM Barrett CL Gao Y Palsson BO The transcription unit architecture of the Escherichia coli genome Nat Biotechnol 2009 27 1043 1049 10.1038/nbt.1582 19881496 PMC3832199

[B2] Albrecht M Sharma CM Reinhardt R Vogel J Rudel T Deep sequencing-based discovery of the Chlamydia trachomatis transcriptome Nucleic Acids Res 2010 38 868 877 10.1093/nar/gkp1032 19923228 PMC2817459

[B3] Mandlik A Livny J Robins WP Ritchie JM Mekalanos JJ Waldor MK RNA-Seq-based monitoring of infection-linked changes in Vibrio cholerae gene expression Cell Host Microbe 2011 10 165 174 10.1016/j.chom.2011.07.007 21843873 PMC3166260

[B4] Liu JM Livny J Lawrence MS Kimball MD Waldor MK Camilli A Experimental discovery of sRNAs in Vibrio cholerae by direct cloning, 5S/tRNA depletion and parallel sequencing Nucleic Acids Res 2009 37 e46 10.1093/nar/gkp080 19223322 PMC2665243

[B5] Perkins TT Kingsley RA Fookes MC Gardner PP James KD Yu L Assefa SA He M Croucher NJ Pickard DJ A strand-specific RNA-Seq analysis of the transcriptome of the typhoid bacillus Salmonella typhi PLoS Genet 2009 5 e1000569 10.1371/journal.pgen.1000569 19609351 PMC2704369

[B6] Passalacqua K Varadarajan A Ondov B Okou D Zwick M Bergman N Structure and complexity of a bacterial transcriptome J Bacteriol 2009 191 3203 3211 10.1128/JB.00122-09 19304856 PMC2687165

[B7] Raghavan R Groisman EA Ochman H Genome-wide detection of novel regulatory RNAs in E. coli Genome Res 2011 21 1487 1497 10.1101/gr.119370.110 21665928 PMC3166833

[B8] Raghavan R Sage A Ochman H Genome-wide identification of transcription start sites yields a novel thermosensing RNA and new cyclic AMP receptor protein-regulated genes in Escherichia coli J Bacteriol 2011 193 2871 2874 10.1128/JB.00398-11 21460078 PMC3133129

[B9] Goldman SR Sharp JS Vvedenskaya IO Livny J Dove SL Nickels BE NanoRNAs prime transcription initiation in vivo Mol Cell 2011 42 817 825 10.1016/j.molcel.2011.06.005 21700226 PMC3130991

[B10] Yoder-Himes D Chain P Zhu Y Wurtzel O Rubin E Tiedje J Sorek R Mapping the Burkholderia cenocepacia niche response via high-throughput sequencing Proc Natl Acad Sci USA 2009 106 3976 3981 10.1073/pnas.0813403106 19234113 PMC2645912

[B11] Livny J Waldor MK Mining regulatory 5′UTRs from cDNA deep sequencing datasets Nucleic Acids Res 2010 38 1504 1514 10.1093/nar/gkp1121 19969537 PMC2836559

[B12] Wang Z Gerstein M Snyder M RNA-Seq: a revolutionary tool for transcriptomics Nat Rev Genet 2009 10 57 63 10.1038/nrg2484 19015660 PMC2949280

[B13] Sorek R Cossart P Prokaryotic transcriptomics: a new view on regulation, physiology and pathogenicity Nat Rev Genet 2010 11 9 16 19935729 10.1038/nrg2695

[B14] Giannoukos G Ciulla DM Huang K Haas BJ Izard J Levin JZ Livny J Earl AM Gevers D Ward DV Efficient and robust RNA-seq process for cultured bacteria and complex community transcriptomes Genome Biol 2012 13 R23 10.1186/gb-2012-13-3-r23 22455878 PMC3439974

[B15] He S Wurtzel O Singh K Froula JL Yilmaz S Tringe SG Wang Z Chen F Lindquist EA Sorek R Hugenholtz P Validation of two ribosomal RNA removal methods for microbial metatranscriptomics Nat Methods 2010 7 807 812 10.1038/nmeth.1507 20852648

[B16] Lennon NJ Lintner RE Anderson S Alvarez P Barry A Brockman W Daza R Erlich RL Giannoukos G Green L A scalable, fully automated process for construction of sequence-ready barcoded libraries for 454 Genome Biol 2010 11 R15 10.1186/gb-2010-11-2-r15 20137071 PMC2872875

[B17] Blencowe BJ Ahmad S Lee LJ Current-generation high-throughput sequencing: deepening insights into mammalian transcriptomes Genes Dev 2009 23 1379 1386 10.1101/gad.1788009 19528315

[B18] Levin JZ Yassour M Adiconis X Nusbaum C Thompson DA Friedman N Gnirke A Regev A Comprehensive comparative analysis of strand-specific RNA sequencing methods Nat Methods 2010 7 709 715 10.1038/nmeth.1491 20711195 PMC3005310

[B19] Parkhomchuk D Borodina T Amstislavskiy V Banaru M Hallen L Krobitsch S Lehrach H Soldatov A Transcriptome analysis by strand-specific sequencing of complementary DNA Nucleic Acids Res 2009 37 e123 10.1093/nar/gkp596 19620212 PMC2764448

[B20] Dornenburg JE Devita AM Palumbo MJ Wade JT Widespread antisense transcription in Escherichia coli MBio 2010 1 1 e00024 10 20689751 10.1128/mBio.00024-10 PMC2912661

[B21] Toledo-Arana A Repoila F Cossart P Small noncoding RNAs controlling pathogenesis Curr Opin Microbiol 2007 10 182 188 10.1016/j.mib.2007.03.004 17383223

[B22] Sharma CM Hoffmann S Darfeuille F Reignier J Findeiss S Sittka A Chabas S Reiche K Hackermuller J Reinhardt R The primary transcriptome of the major human pathogen Helicobacter pylori Nature 2010 464 250 255 10.1038/nature08756 20164839

[B23] Lasa I Toledo-Arana A Dobin A Villanueva M de los Mozos IR Vergara-Irigaray M Segura V Fagegaltier D Penades JR Valle J Genome-wide antisense transcription drives mRNA processing in bacteria Proc Natl Acad Sci USA 2011 108 20172 20177 10.1073/pnas.1113521108 22123973 PMC3250193

[B24] Nicolas P Mader U Dervyn E Rochat T Leduc A Pigeonneau N Bidnenko E Marchadier E Hoebeke M Aymerich S Condition-dependent transcriptome reveals high-level regulatory architecture in Bacillus subtilis Science 2012 335 1103 1106 10.1126/science.1206848 22383849

[B25] Raghavan R Sloan DB Ochman H Antisense transcription is pervasive but rarely conserved in enteric bacteria MBio 2012 3 4 e00156 12 22872780 10.1128/mBio.00156-12 PMC3419515

[B26] Livny J Teonadi H Livny M Waldor MK High-throughput, kingdom-wide prediction and annotation of bacterial non-coding RNAs PLoS One 2008 3 e3197 10.1371/journal.pone.0003197 18787707 PMC2527527

[B27] Fozo EM Kawano M Fontaine F Kaya Y Mendieta KS Jones KL Ocampo A Rudd KE Storz G Repression of small toxic protein synthesis by the Sib and OhsC small RNAs Mol Microbiol 2008 70 1076 1093 10.1111/j.1365-2958.2008.06394.x 18710431 PMC2597788

[B28] Kim KS Pelton JG Inwood WB Andersen U Kustu S Wemmer DE The Rut pathway for pyrimidine degradation: novel chemistry and toxicity problems J Bacteriol 2010 192 4089 4102 10.1128/JB.00201-10 20400551 PMC2916427

[B29] Quentmeier A Holzenburg A Mayer F Antranikian G Reevaluation of citrate lyase from Escherichia coli Biochim Biophys Acta 1987 913 60 65 10.1016/0167-4838(87)90232-9 3555623

[B30] Metcalf WW Wanner BL Involvement of the Escherichia coli phn (psiD) gene cluster in assimilation of phosphorus in the form of phosphonates, phosphite, Pi esters, and Pi J Bacteriol 1991 173 587 600 1846145 10.1128/jb.173.2.587-600.1991 PMC207049

[B31] Tarazona S Garcia-Alcalde F Dopazo J Ferrer A Conesa A Differential expression in RNA-seq: a matter of depth Genome Res 2011 21 2213 2223 10.1101/gr.124321.111 21903743 PMC3227109

[B32] Anders S Huber W Differential expression analysis for sequence count data Genome Biol 2010 11 R106 10.1186/gb-2010-11-10-r106 20979621 PMC3218662

[B33] Cheung AL Eberhardt KJ Fischetti VA A method to isolate RNA from gram-positive bacteria and mycobacteria Anal Biochem 1994 222 511 514 10.1006/abio.1994.1528 7532381

[B34] Fisher S Barry A Abreu J Minie B Nolan J Delorey TM Young G Fennell TJ Allen A Ambrogio L A scalable, fully automated process for construction of sequence-ready human exome targeted capture libraries Genome Biol 2011 12 R1 10.1186/gb-2011-12-1-r1 21205303 PMC3091298

[B35] Li H Durbin R Fast and accurate short read alignment with Burrows-Wheeler transform Bioinformatics 2009 25 1754 1760 10.1093/bioinformatics/btp324 19451168 PMC2705234

[B36] Gardner PP Daub J Tate JG Nawrocki EP Kolbe DL Lindgreen S Wilkinson AC Finn RD Griffiths-Jones S Eddy SR Bateman A Rfam: updates to the RNA families database Nucleic Acids Res 2009 37 D136 D140 10.1093/nar/gkn766 18953034 PMC2686503

