# Supplementary figures and images for: How deep is deep enough for RNA-Seq profiling of bacterial transcriptomes?
Source: BMC Genomics. 2012 Dec 27;13:734. doi: 10.1186/1471-2164-13-734 (PMC3543199; doi:10.1186/1471-2164-13-734)

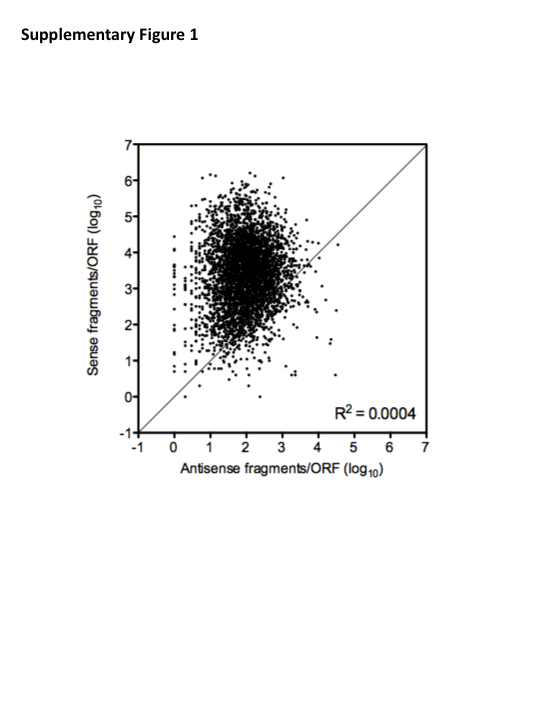

Supplement: Additional file 3 — Figure S1. Correlation of coverage of the sense and antisense strands of annotated ORFs. [file 1471-2164-13-734-S3.tiff]
